# Rapid Losses of Surface Elevation following Tree Girdling and Cutting in Tropical Mangroves

**DOI:** 10.1371/journal.pone.0107868

**Published:** 2014-09-22

**Authors:** Joseph Kipkorir Sigi Lang'at, James G. Kairo, Maurizio Mencuccini, Steven Bouillon, Martin W. Skov, Susan Waldron, Mark Huxham

**Affiliations:** 1 Kenya Marine and Fisheries Research Institute, Mombasa, Kenya; 2 School of Life, Sport and Social Sciences, Edinburgh Napier University, Edinburgh, United Kingdom; 3 School of Geosciences, University of Edinburgh, Crew Building, Edinburgh, United Kingdom; 4 Department of Earth and Environmental Sciences, Katholieke Universiteit Leuven, Leuven, Belgium; 5 School of Ocean Sciences, Bangor University, Menai Bridge, Anglesey, United Kingdom; 6 School of Geographical and Earth Sciences, University of Glasgow, Glasgow, South Lanarkshire, United Kingdom; 7 Centre for Ecological Research and Forestry Applications, Universitat Autònoma de Barcelona, Barcelona, Spain; Centro de Investigacion Cientifica y Educacion Superior de Ensenada, Mexico

## Abstract

The importance of mangrove forests in carbon sequestration and coastal protection has been widely acknowledged. Large-scale damage of these forests, caused by hurricanes or clear felling, can enhance vulnerability to erosion, subsidence and rapid carbon losses. However, it is unclear how small-scale logging might impact on mangrove functions and services. We experimentally investigated the impact of small-scale tree removal on surface elevation and carbon dynamics in a mangrove forest at Gazi bay, Kenya. The trees in five plots of a *Rhizophora mucronata* (Lam.) forest were first girdled and then cut. Another set of five plots at the same site served as controls. Treatment induced significant, rapid subsidence (−32.1±8.4 mm yr^−1^ compared with surface elevation changes of +4.2±1.4 mm yr^−1^ in controls). Subsidence in treated plots was likely due to collapse and decomposition of dying roots and sediment compaction as evidenced from increased sediment bulk density. Sediment effluxes of CO_2_ and CH_4_ increased significantly, especially their heterotrophic component, suggesting enhanced organic matter decomposition. Estimates of total excess fluxes from treated compared with control plots were 25.3±7.4 tCO_2_ ha^−1^ yr^−1^ (using surface carbon efflux) and 35.6±76.9 tCO_2_ ha^−1^ yr^−1^ (using surface elevation losses and sediment properties). Whilst such losses might not be permanent (provided cut areas recover), observed rapid subsidence and enhanced decomposition of soil sediment organic matter caused by small-scale harvesting offers important lessons for mangrove management. In particular mangrove managers need to carefully consider the trade-offs between extracting mangrove wood and losing other mangrove services, particularly shoreline stabilization, coastal protection and carbon storage.

## Introduction

Mangrove forests are highly productive systems and often allocate a large proportion of their energy budget to root production [1]–[3]. Because of the presence of aerial roots, mangroves trap allochthonous organic matter in sediment, with carbon (C) sequestration rates exceeding those of terrestrial tropical forests by a factor of ∼6 [4]. Unlike terrestrial forest soils, mangrove sediments do not attain C saturation because of continued sediment accumulation and vertical accretion [5] and hence the size of the C store continues to increase over time [6]. Anoxia, low levels of nutrients and the high lignin content of the roots result in slow decomposition of below-ground organic matter [7]–[9] and the accumulation of large reserves of peat and C-rich sediments [10]–[12]. Mangroves are thus amongst the most carbon dense of all forests, with C stocks sometimes exceeding 1000 tonnes C ha^−1^ [12]–[14], and hence play an important role in global carbon storage [15]–[17].

As a result of continued vertical accretion and below-ground root growth, surface elevation of mangroves increases over time at rates of up to 4.8 mm yr^−1^ e.g. [5]. These increases in surface elevation are important in allowing mangrove recovery after natural disturbances and are considered essential for many mangroves to survive projected sea level rise of 1.7 to 3.3 mm yr^−1^ this century [18], [19].

Human disturbances such as wood harvesting and clearing threaten to impair these important ecological processes. Trends in mangrove loss are alarming, with an estimated 30–50% of forests lost over the past half century [20], [21]. Although rates of loss may be declining [22] they remain high, typically 0.7–3% y^−1^, partly because of high levels of poverty and dense human populations along many tropical coasts [13]. While a recent estimate of the impact of such losses on the total mangrove carbon sink suggested that mangrove destruction could contribute up to 10% of the annual GHG emissions from land use change [12], little understanding exists of the impact of mangrove harvesting on surface elevation dynamics. Reductions in surface elevation after harvesting may be caused by a combination of processes. Following harvesting, root growth and expansion stops, whilst decomposition of dead roots and old organic matter may be accelerated by higher temperatures in the exposed substrate and increased sediment oxidation. Sediment erosion may also increase and the lack of aerial roots may prevent continued accumulation of allochthonous sediment. Additionally, the aerenchymatous tissues of the dying roots may shrink leading to increased bulk density but lower elevation. Finally, leaching of dissolved inorganic and organic C and lateral transport to the sea may occur [2], [3], [17], [23], [24].

Few studies have directly measured the impacts of tree removal on below-ground carbon storage and surface elevation. Two studies report on the effects of hurricanes [25], [26] and one on total deforestation [27]. Under such extreme conditions carbon losses can be large with resulting ‘peat collapse’ and coastal erosion. The impacts of smaller scale tree loss are even less well known, although work from Micronesia after non-experimental tree felling [28] and Florida following lightning strikes [29] shows that surface elevation losses might be rapid. Whilst healthy forests are likely to be resilient and show recovery, those under anthropogenic pressure may experience longer term change. Mangroves of the Western Indian Ocean (WIO) region experience small-scale but widespread degradation from indiscriminate harvesting [30], [31]. A recent assessment of mangrove decline in Kenya has indicated an annual loss of 0.7%, which underestimates the anthropogenic stressors since it records only total canopy removal [32]. Here we document the first controlled experiment testing the impacts of small-scale cutting, the most common form of mangrove exploitation in the WIO region, on sediment carbon losses and surface elevation. Increasing attention is being paid to avoiding deforestation by Reducing Emissions from Deforestation and Degradation and conservation of forest ecosystems (REDD+) schemes and to managing forests for a range of services, not only wood production. It is essential therefore that the impacts of forest management scenarios on key ecosystem services such as coastal protection and carbon sequestration are analysed to understand possible trade-offs between these and extractive uses.

Mangrove forests of the WIO region are utilized by the local communities for construction and fuelwood [33]. In Kenya mangroves are the only natural forests currently licensed by the Kenya Forest Service (KFS) for wood harvesting [30], [34]. In combination with widespread illegal harvesting this has left many mangrove areas either degraded or completely denuded of vegetation [30], [35]. Since mangroves meet ∼70% of the wood requirements of the coastal population [36], there is always a ready market for mangrove poles, especially in major coastal towns.

We used a controlled experiment to explore the impacts of tree harvesting on: a) surface elevation dynamics; b) the main processes affecting changes in surface elevation i.e., sediment accretion, sediment properties such as bulk density, %C and sediment moisture, and organic matter decomposition; and c) sediment surface C efflux in a natural mangrove forest, here at Gazi bay, Kenya. The C flux from forest floors comes from root (autotrophic) respiration and sediment/soil organic matter decomposition (heterotrophic respiration) [37]. The relative contributions of these sources have not been distinguished in mangroves; such partitioning is useful in understanding carbon stocks and flows in forests. Girdling trees stops the flow of photosynthates to the root system and thereby stops metabolic activities in the roots whilst maintaining the tree canopy. Hence it can be used to separate the components of soil respiration, since CO_2_ emitted from the sediment shortly after girdling is assumed to be primarily from organic matter decomposition e.g., [38]–[40]. Here we also report on the first time this approach has been used for mangroves.

## Results

### Surface elevation and sediment accretion

**Figure 1:**
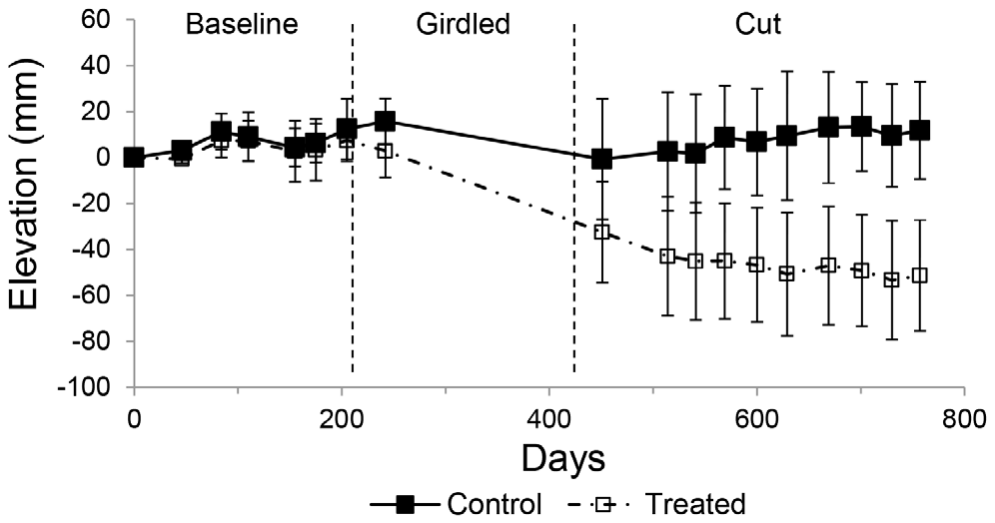
Trends in surface elevation change in control and treated sites in *R. mucronata* forest at Gazi bay, Kenya. Error bars are 95% CI. Vertical broken lines indicate periods when trees were girdled and cut in the treatment plots. Baseline, girdled and cut periods ran from March 2009 to October 2009 (205 days), December 2009 to May 2010 (189 days) and May 2010 to April 2011 (343 days), respectively. The controls and the treatment consisted of five replicates each.

At the conclusion of monitoring after 760 days the control plots had gained mean surface elevation of +11.1±10.5 mm, at a mean rate of +4.2±1.4 mm yr^−1^, while the treatment plots showed a subsidence of −51.3±12.0 mm, at a mean rate of −32.1±8.4 mm yr^−1^ (Figure 1). For a period of 110 days after setting up the horizon marker, both control and treated plots experienced similar trends in sediment accretion, ranging from 5.5 to 8.0 mm (controls, mean 6.4±1.4 mm) and 2.5 to 11.7 mm (treated, mean 5.2±4.6 mm). Disturbance of the horizon marker by crab activities in the control plots did not allow further monitoring beyond four months after set up.

### Sediment Surface Carbon Fluxes

**Figure 2:**
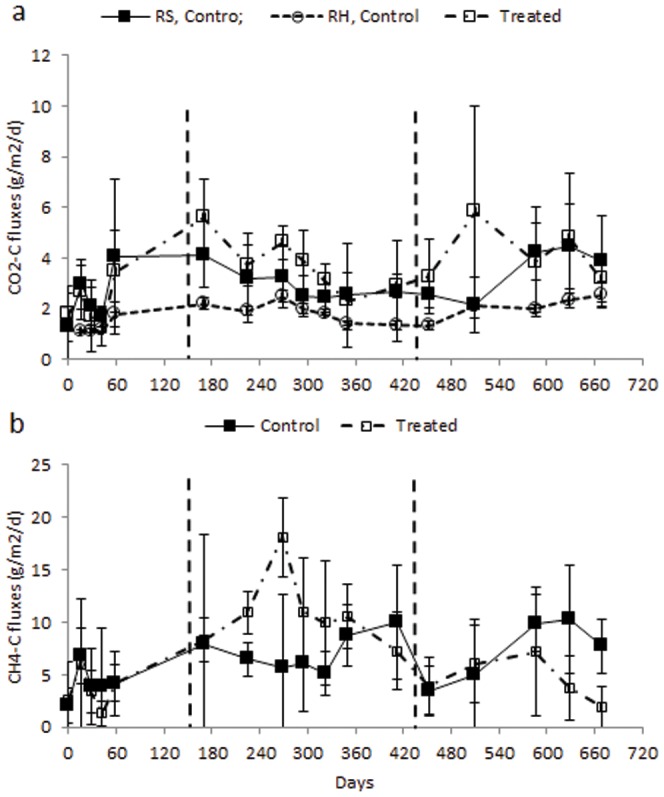
Mean (±95% CI) Carbon emissions. a) CO_2_ fluxes of total sediment respiration (R_S_) (solid line with filled squares), heterotrophic respiration (R_H_) (dashed line with open circles) in control plots and CO_2_ fluxes from treated plots (broken line with open squares) and b) CH_4_ emissions in *R. mucronata* forest at Gazi bay Kenya. Vertical broken lines indicate periods when trees were girdled and cut in the treatment plots. Sampling for baseline, girdled and cut periods were done from June 2009 to August 2009 (84 days), December 2009 to May 2010 (189 days) and May 2010 to April 2011 (343 days), respectively. The controls and the treatment consisted of five replicates each.

**Figure 3:**
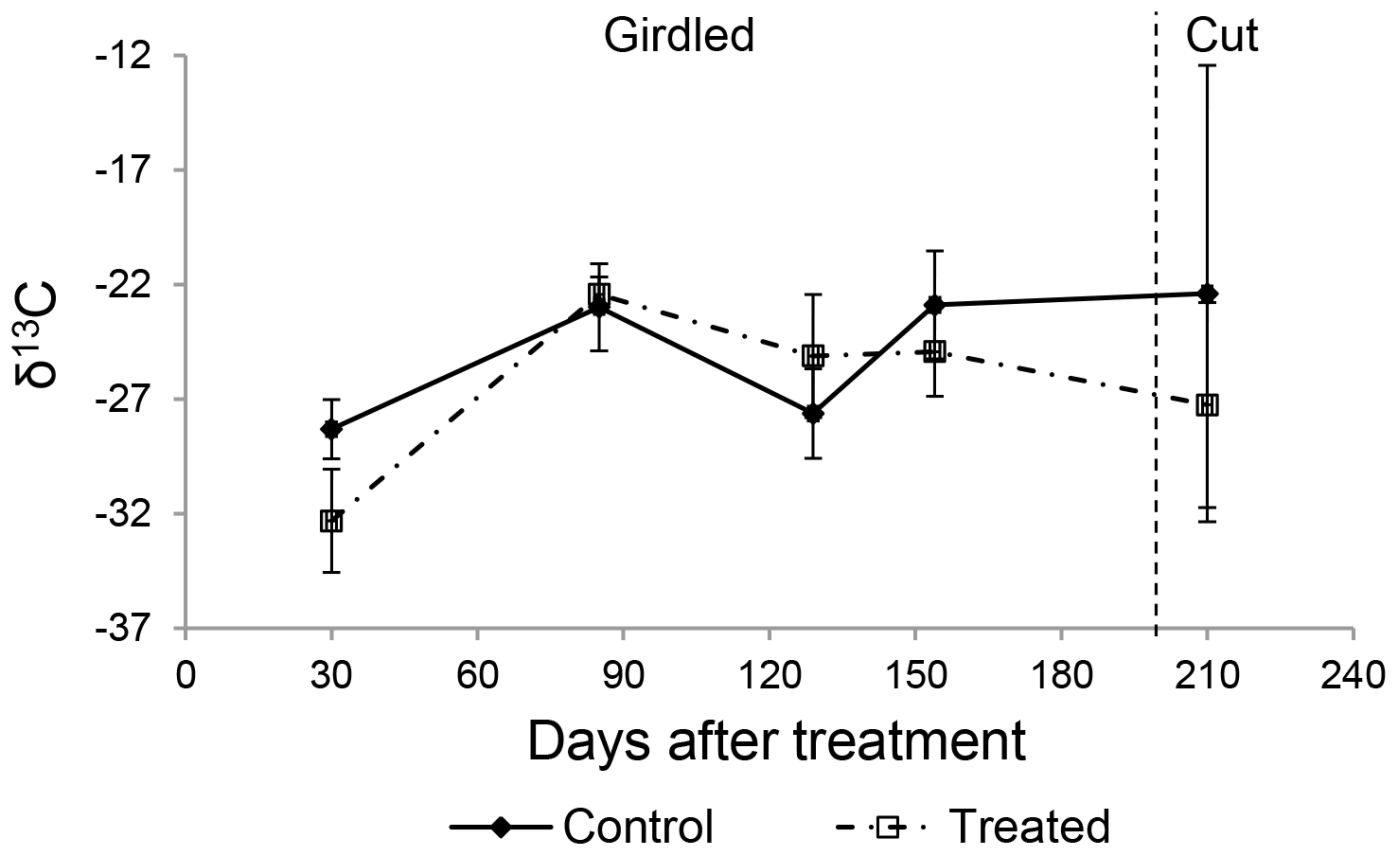
Trends in δ^13^C of sediment respired CO_2_ in control and treated sites in *R. mucronata* forest at Gazi bay, Kenya. Values are means ±95% CI. Vertical broken line indicates when the trees were clear-cut in treatment plots. The controls and the treatment consisted of five replicates each.

Approximately 30 days after girdling, CO_2_-C emissions in treated plots increased and remained higher than in controls throughout the girdled period (Table 1 and Figure 2). For the first two months after cutting, CO_2_-C emissions in the treated plots were similar to controls, but then increased again for three months before dropping to levels similar to the controls by the end of the sampling period (3.2±0.9 vs. 3.9±1.8 gCO_2_–C m^−2^ d^−1^, respectively; Figure 2). At ∼30 days after girdling, the δ^13^C signature of the sediment respired CO_2_ from the treated plots was significantly more ^13^C-depleted than in the controls (Figure 3). Methane emissions were highly variable and increased in the treated plots during the girdled period only (Figure 2). Mean emissions of both CO_2_–C and CH_4_–C were significantly higher in treated than control plots during the girdled period (Table 1). Throughout the treatment period, the mean sediment temperature in the treated plots was higher than the control plots by 0.9 to 5.8°C.

**Table 1:** Nested design ANOVA for carbon fluxes in *R. mucronata* secondary forest, with mean data for each of six chambers per plot nested within treatment; the data for CO_2_ were log-transformed.

Period	Variable	Source of Variation	DF	MS	F	P
Girdled	CO_2_	Temperature	1	0.017	0.420	0.518
		Burrows	1	0.017	0.45	0.506
		Treatment	1	0.152	4.73	0.036
		Plot (Treatment)	8	0.025	0.64	0.736
		Error	48	0.039		
	CH_4_	Temperature	1	46.140	2.4	0.128
		Burrows	1	2.660	0.14	0.712
		Treatment	1	745.800	20.04	0.000
		Plot (Treatment)	8	55.700	2.9	0.01
		Error	48	19.230		
Clear-cut	CO_2_	Temperature	1	0.005	0.16	0.695
		Burrows	1	0.044	1.26	0.267
		Treatment	1	0.001	0.02	0.897
		Plot (Treatment)	8	0.028	0.81	0.6
		Error	48	0.035		
	CH_4_	Temperature	1	24.758	3.43	0.07
		Burrows	1	5.73	0.79	0.378
		Treatment	1	1.458	0.19	0.667
		Plot (Treatment)	8	10.834	1.5	0.182
		Error	48	7.225		

### Separation of sediment autotrophic and heterotrophic respiration components

Girdling was not effective in separating the components of sediment respiration since the girdled trees began to lose leaves and die within one month of girdling; this was much faster than recorded for terrestrial trees e.g., [40]. Therefore, an indirect regression method of estimating autotrophic respiration was used [38]. In each of the control plots, live root biomass was determined after the final sampling in April 2011 (530 days after treatment) by excavations made beneath the pre-marked positions of each chamber. The regression of CO_2_ fluxes, measured at the final sampling for each control plot chamber, against the live root biomass and sediment surface temperature adjacent to each chamber, was significant:

Multiple regression: lnR_S_ = −3.093+0.0002*live root biomass +0.127*temperature; R^2^ = 0.37; P = 0.044, 0.011 and 0.012 for the constant, live root biomass and sediment temperature, respectively.

Heterotrophic respiration (R_H_) for final sample values was calculated by applying the equation above, while setting the value of root biomass to zero [38]. Autotrophic respiration (R_A_), obtained by subtracting the values for R_H_ from the measured total sediment respiration (R_S_), contributed a mean (±95% CI) of 41.5±11.8% to R_S_ at the final sampling. Across the entire sampling period, R_A_ contributed an average of 40.5±7.0% to R_S_, which was not significantly different from that obtained at the final sampling (t-test, t = −0.16, P = 0.874). The partitioning of the components of total R_S_ in the control plots allowed a comparison of the treated plot CO_2_ fluxes (where autotrophic R was zero by definition) and control plot R_H_. Sediment respiration of the treated plots was higher than control plots R_H_ throughout the treatment period (during both girdling and cutting) by 0.6 to 3.7 g CO_2_–C m^−2^ d^−1^ (Figure 2).

### Estimated C losses from the sediment

Combining data on elevation changes with sediment characteristics provided one estimate of total carbon losses of 35.7±76.9 t C ha^−1^ from the treated compared to control plots.

The net belowground carbon losses due to treatment estimated from sediment surface flux data amounted to 14.2±10.3 tCO_2_ ha^−1^ (mean rate of 9.8±7.1 t CO_2_ ha^−1^ yr^−1^) over a period of 530 days after treatment. When only R_H_ in control plots was considered, the net additional C loss amounted to 36.7±10.7 t CO_2_ ha^−1^, with a mean rate of 25.3±7.4 t CO_2_ ha^−1^ yr^−1^.

### Root Decomposition

After 267 days root-bags in the treated plots had lost significantly more mass than those in the controls, with rates of 0.19±0.02 and 0.16±0.03% dry weight loss day^−1^, respectively (t-test, t = −2.06, P = 0.049). The trend in decomposition rate in the treated plots indicated that it was increasing with time, while the rate in the control plots remained constant between 156 and 267 days.

### Belowground Biochemical Characteristics

The plant roots had a similar δ^13^C signature to that of the sediment carbon (Table 2). Treated plots showed significant reductions in % C and sediment moisture and significant increases in bulk density. The sediment carbon stocks to a depth of 1 m ranged from 414.1 to 610.7 t C ha^−1^ for controls and 457.3 to 586.3 t C ha^−1^ for treated plots. Overall the control plots tended to have higher mean C stocks than treated plots, with mean (±95% CI) of 524.1±88.8 vs. 488.4±68.6 tC ha^−1^, respectively, but the difference was not significant due to the very large small-scale variability in sediment properties.

**Table 2:** Belowground roots and sediment biochemical characteristics in control and cut plots in *R*.*mucronata* forest at Gazi bay, Kenya.

Variable	Control	Cut
% OC_R_	37.2±1.7	-
δ^13^C_R_	−27.0±0.3	-
Sediment moisture content (%)	46.5±4.0	42.0±5.7
Bulk Density (g cm^−3^)	0.84±0.08	0.88±0.10
Sediment C concentration (g C cm^−3^)	0.052±0.008	0.052±0.008
% N	0.38±0.04	0.36±0.11
TOC/TN	18.59±0.8	17.6±0.7
δ^13^C_S_	−27.2±0.2	−27.4±0.1
Sediment C stocks* (t C ha^−1^)	524.1±62.7	488.4±48.4

Values are means ±95% CI, OC_R_ =  organic carbon content of mangrove roots, δ^13^C_R_ and δ^13^C_S_ =  carbon isotopic value of mangrove roots and sediment organic matter, respectively, and TOC and TN =  total carbon and nitrogen content of the sediment organic matter, respectively.

*Sediment C stocks to 1 m depth.

## Discussion

Tree death induced rapid and significant subsidence. This was likely due to rapid decomposition of fine roots exacerbated by the absence of new root growth, as shown by the significant difference in root bag decomposition. In addition, sediment compaction and the collapse of arenchymatic tissues due to consolidation of air spaces (as shown by the increase in bulk density), and the significant loss of sediment moisture caused by the treatment, may also have contributed. Rates of sediment accretion did not differ significantly between control and treated plots hence the subsidence was not due to enhanced erosion. The rate of subsidence was surprisingly high given the relatively small scale of the treatments; massive hurricane damage, which led to ‘peat-collapse’ in Honduran mangroves, caused elevation losses only around ∼0.3 as fast as those recorded here (−11 compared with −32 mm yr^−1^ [25]).

Interestingly, the C loss of around 25.3±7.4 t CO_2_ ha^−1^ yr^−1^ reported here due to small-scale cutting was similar to that reported for mangrove forests impacted by large-scale clearing (29 tCO_2_ ha^−1^ yr^−1^) [27], and that inferred from peat collapse due to hurricane damage (15 t CO_2_ ha^−1^ yr^−1^) [25]. Much of the C loss occurred within the first year after treatment (mean ±SD? rates of 13.22±9.71 and 7.86±6.77 t CO_2_ ha^−1^ yr^-1^ during the girdled and cut periodsrespectively) and by ∼1.5 years the C losses induced by treatment began to drop. A similar pattern was recorded in clear-cut mangroves in Belize in which the C emissions in disturbed areas declined with time [27]. However, there was evidence in our study that decomposition of sediment organic carbon (SOC) - not only newly-killed root material - was enhanced by treatment, and that rates of SOC decomposition might be increasing with time. Buried root bags recorded significantly higher rates of decomposition in treated plots (0.19±0.02 vs. 0.16±0.02% dry weight loss day^-1^, respectively) with most of the difference occurring after the first set of root bags were retrieved (150 days after burial). This was probably due to enhanced sediment surface temperatures in the cut plots due to canopy removal, since the treated plots experienced increases in sediment surface temperatures of 0.8 to 5.9°C compared to the control plots. Therefore, these results highlight the potential impact of physico-chemical changes on C losses in cut forests, which are separate from and additional to the losses from root death per se.

Although our estimates suggest high rates of below-ground C loss caused by tree death (similar to those seen following much larger impacts such as hurricanes) they are likely to represent an underestimate, since surface fluxes of CO_2_ cannot account for below-ground, lateral flows of carbon in dissolved inorganic carbon (DIC) [2], [3], [24].

CH_4_ emissions were significantly enhanced during the girdled period, possibly because of the addition of easily fermentable substrates [41]–[43] from dying roots. However, emissions in both treatment periods (girdled and cut respectively 0.7–1.5 and 0.2–0.9 mmol m^−2^ d^−1^), did not exceed those reported for a number of pristine mangrove forests worldwide, 0.01–5.0 mmol m^−2^ d^−1^ [40]. Sediment-respired CO_2_ collected shortly after girdling showed significant ^13^C-depletion (−32.3 ‰; Fig. 3). Such depleted signatures are unlikely to arise from respiration of existing organic matter alone, as here δ^13^C would be similar to the control. Rather, as coincident with increased CH_4_ emissions, the most parsimonious explanation is that oxidation of methane comprised a component of the CO_2_ efflux. Assuming a typical δ^13^C value for methane in marine environments of −60 ‰ [44], the measured ^13^C-depletion would represent an additional 12% CO_2_ contribution from methane oxidation.

Overall, our experiment showed that small-scale harvesting for wood production as is typical in the countries around the Western Indian Ocean caused significant sediment subsidence, which was caused primarily by sediment compaction, loss of sediment moisture and increased organic matter decomposition (both aerobic and anaerobic). Study of lightning damage in Florida mangroves, creating forest gaps larger than our experimental plots, showed even more subsidence (of up to 61 mm), but also suggests that undisturbed forests can recover [29]. Such evidence should not induce complacency since pressures on mangroves in many areas are increasing. Sea level is projected to rise this century [18], with current projections varying between 1.7 and 3.3 mm yr^−1^ [19]. Employing the lower estimate of sea level rise of 1.7 mm yr^−1^, and the conservative assumption that our cumulative surface elevation loss of 62.4 mm will not increase further in the future, the impact of the loss we observed is equivalent to increasing sea level rise for ∼37 years. Hence our study raises serious concerns that the combination of effects caused by global change and small-scale forest harvesting on mangrove surface elevation may be highly damaging to these sensitive ecosystems.

## Conclusions

Kenyan mangroves are being lost at a rate of 0.7% cover per year [45], mainly because of the demands of the large and growing population for wood fuel and timber [34], [36].

A new approach towards the sustainable management of mangrove forests uses payments for ecosystem services (PES) schemes such as the proposed REDD+ programme [46]. Such schemes should allow combining income from provisioning and regulating ecosystem services (such as timber and coastal protection or carbon sequestration), but only if the trade-offs between them are understood and managed. The present work demonstrates the susceptibility of mangroves to rapid subsidence (with consequent enhanced vulnerability to sea level rise and erosion) and at least short-term carbon loss following relatively small-scale and controlled canopy removal. With this understanding, management regimes aiming to conserve carbon stocks and promote climate resilience should be wary of clear-cutting, particularly in areas that may be exposed to erosion, and should emphasise instead selective cutting, rapid replacement of the lost canopy and the maintenance of un-cut buffer strips on seaward fringes to avoid the risks of erosion.

## Materials and Methods

### Study site

The study was carried out at Gazi Bay (4^o^ 25′ S and 4^o^ 27′ S; 39^o^ 50′ E and 39^o^ 50′ E), ∼55 km south of Mombasa, Kenya. Gazi Bay is a creek system with a total area of 615 ha mangrove forest [47], dominated by *Rhizophora mucronata* (Lam), *Ceriops tagal* (Perr.) C. B. Robinson and *Avicennia marina* (Forsk.) Vierh. The mean annual precipitation of Gazi bay ranges from 1000–1600 mm [47]. The bay receives freshwater from two semi-permanent rivers: Kidogoweni to the north, which discharges in to the Kidogoweni creek, and Mkurumuji river to the south, discharging to the mouth of the bay. The forest is government-owned and permission to use the site was granted by the Kenya Marine and Fisheries Research Institute; our experiments did not affect endangered or protected species, and the individual featured in the accompanying image gave written informed consent (as outlined in the PLOS consent form) to publish this image.

### Experimental Design

Five pairs of 12 m×12 m plots were established in March 2009 at high water mark (height above mean sea-level: 2.91±0.01 m) within a *Rhizophora mucronata* dominated forest (specific location: 4°24′32″S 39°31′23″E). In October 2009, five plots (one from each pair) were randomly allocated to ‘treatment’ and all the trees within them were girdled at ∼20 cm above the highest prop root. Girdling is a method that has been used in a number of terrestrial forests to estimate the contribution of root respiration to total sediment gas flux. The rationale is to prevent the flow of carbohydrates from the tree canopy to their roots (thus stopping root respiration) whilst leaving the above-ground components relatively undisturbed; trees may retain foliage for many months after girdling [39], [40]. The other plots in the five pairs served as the controls. In May 2010, all the trees in the girdled plots were cut at ∼20 cm above the highest stilt roots and all the debris, excepting small fragments, was removed. The treated plots were allowed to stabilize after disturbance for approximately three weeks; thereafter sampling was resumed. During each treatment operation saplings and seedlings were cut down. Hence the experiment consisted of three sampling periods: a) baseline (pre-treatment) (June 2009 to August 2009, 84 days), b) girdled period (December 2009 to May 2010, 159 days) and c) cut period (May 2010 to April 2011, 343 days).

### Surface elevation and sediment accretion

Surface elevation dynamics were monitored using surface elevation stations, consisting of two stainless steel rods (6 mm by 1 m) and a horizon marker (kaolin) set up in a 20 cm×20 cm quadrat in each plot. The rods were installed leaving a height of 20 cm above the ground and at opposite corners of the quadrat such that measurements would be made diagonally across it. Height measurements vertically from the ground surface to the heights of the rods were made at seven clearly marked points along a wooden board placed across the rods. Subsequent measurements were made at the same points along the wooden board. All data were averaged to give a single measurement per plot per time. Sediment accretion was determined from measurements of height above the horizon marker. In each quadrat, at least four sediment blocks of 2 cm×2 cm were carefully removed with a sharp knife, the height of sediment above the horizon marker was noted and the block was then carefully replaced in its original position. This approach allows the separation of total surface elevation/subsidence (which depends on both the accretion or erosion of new sediments and on below-ground processes such as root growth and expansion) from accretion/erosion [25].

Samples for sediment physico-chemical analysis were taken from each plot during February 2010 and August 2012. A sediment core was taken in the centre of the plot using a plastic corer (diameter 6 cm, length 3 m) in February 2010 and again in August 2012. Subsamples of this large core were taken with a small stainless steel corer (diameter 3 cm, length 5 cm) at depths of 0, 2, 4, 8, 10, 20, 30 and 40 cm (February 2010) and depths of 0, 10, 20, 30, 50 and 100 cm (August 2012). To minimize compression of the sediment the coring was done in a series of stages according to the depth profiles for sub-sampling. Sediment samples were oven-dried at 80°C to constant dry weight and bulk density was determined at six depths down to 1 m. The oven-dried samples were transferred to the Department of Earth and Environmental Sciences, KU Leuven, Belgium for analysis of % OC (organic carbon), % N and δ^13^C of OC. The concentrations of OC, total N, and δ^13^C values of sediment OC were measured on subsamples weighed into Ag cups, acifidied with dilute HCl to remove inorganic C, and analysed with a Thermo Flash HT elemental analyser coupled to a Thermo Delta V Advantage IRMS (Conflo IV interface). Data were calibrated with IAEA-C6, and internally calibrated acetanilide and leucine. From the sediment bulk density and OC content, the carbon density and hence the sediment carbon stocks down to 100 cm were derived for the control and treated plots.

### Gas fluxes and stable carbon isotope signatures

Gas flux (CO_2_, CH_4_) samples were collected at approximately monthly intervals at low tide during spring tides using six chambers per plot; some sampling times that were missed due to loss or damage to bags and other equipment. Each chamber was inserted ∼5 cm in to the sediment, occupying an area of 0.064 m^2^ with an internal volume of 0.011 m^3^. The samples from each chamber were taken 20 minutes after closure. Using a 60 ml syringe, at least 240 ml of gas were transferred from each chamber to labelled airtight gas-bags (Cali-5-bond gas bags, Calibrated Instruments Inc. USA). A gas sample of ambient concentration was taken from each chamber before closure; ambient air concentration samples for each plot were collected in one gas bag. Linearity checks were performed by repeatedly sampling the chamber gas for periods of about 60 minutes. They showed that a linear approximation over a 20 minutes period resulted in ∼15% underestimation of the slope of gas concentration increase over time. This systematic downward bias was not corrected for. Sediment surface temperature measurements were made beside each chamber with a temperature probe inserted to ∼1 cm in to the sediment. The number of crab burrows within the area enclosed by the chamber was noted. The positions of the chambers were marked for subsequent sampling; chambers were always returned to the same sampling positions within plots. Samples for δ^13^C analysis of CO_2_ were transferred from the chambers to 12 ml pre-evacuated exetainers (Labcoexetainer, Labco Ltd., High Wycombe, UK). Gas flux samples were analysed at the Institute of Atmospheric and Environmental Sciences, University of Edinburgh, UK. For CO_2_, the samples were analysed by gas chromatography (GC) using a Perkin Elmer Model 310 with a thermal conductivity detector (TCD). Concentrations of CH_4_ were measured using GC (Hewlett Packard 5890 GC, Hewlett Packard Ltd, Stockport, Cheshire, UK) equipped with a flame ionisation detector (FID) and a digital integrator. The δ^13^C analysis samples were transferred to the Department of Earth and Environmental Sciences, KU Leuven, Belgium and analysed using a Sercon 20–20 isotope ratio mass spectrometer (IRMS) interfaced with a cryofocussing unit.

In May 2011, at the end of the experiment, all roots beneath each chamber in the control plots were excavated to a depth of 60 cm, washed of sediments and separated in to live root and dead plant materials. Dead roots were differentiated based on the loss of structural integrity, colour and signs of decomposition [5]. The samples were oven-dried at 80°C before weighing.

### Decomposition in Root-bags

In September 2010, live roots were excavated within the *R*. *mucronata* forest contiguous to the experimental plots. Nylon mesh (1 mm) bags each containing ∼30 g fresh roots were buried to ∼20 cm depth at six random points within each plot. Three bags were retrieved from each plot 156 days after burial, whilst the other three were retrieved 267 days after burial. The contents of each root-bag were rinsed and oven-dried at 80°C for 24 h before weighing. The rate of root decay (% weight loss day^−1^) was calculated as the % weight loss divided by the number of days buried, using wet-dry weight conversion factors derived from representative samples of live roots, oven-dried at 80°C until constant dry weight.

### Statistical analysis

The data for CO_2_ were log-transformed and the analysis for each gas was executed using MINITAB 14 software package. Initial analyses included time in repeated measures models, however there were multiple significant interactions preventing legitimate conclusions and so data were separated into the three experimental periods. The gas flux values for each chamber were pooled across each period for the controls and treated plots, giving six single values per period for each plot. For each period, nested ANOVA was carried out for each gas, with variation among plots (i.e. 6 chambers) nested within treatment and sediment surface temperature and crab burrows as covariates. Estimates for respired δ^13^C were derived from the Miller-Tans mixing model combined with geometric regression [48], [49]. Kayler et al., [49] found that the combination of geometric regression and Miller-Tans mixing model gave the most accurate and precise estimate of δ^13^C_S_ (S =  sediment respired CO_2_). The gas mixing models are based on the conservation of mass given as [48]: 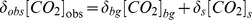



This equation describes the gas observed (obs) as coming from two sources: background atmosphere (bg) and source of respiration (s), where δ refers to the isotopic value of each component. Details of the Miller-Tans mixing model combined with geometric regression are discussed by Kayler *et al*., [49]. The mean δ^13^C of the respired CO_2_ for each plot was analysed using two-sample t-tests.

To examine the autotrophic contribution to sediment fluxes, stepwise multiple regressions (forward and backward elimination) were performed, with the final CO_2_ fluxes measured in each control chamber in April 2011 as the dependent variable and the live root biomass, sediment surface temperature and crab burrows for each chamber as the independent variables. The equation takes the form of y = k+a*roots +b*temperature + c*burrows; where *k* =  constant, *a*, *b* and *c* are the coefficients of the estimators. The number of crab burrows was not significant, and hence this term was omitted from the equation. The significant factors were used in estimating the autotrophic respiration from the final CO_2_ flux data. First, the heterotrophic respiration (R_H_) was calculated as the value of the ‘*y*’ when live root biomass  = 0, i.e. R_H_ = k+ b*temperature. Then the autotrophic respiration (R_A_) was obtained as the difference between total sediment respiration (R_S_) and the heterotrophic component (R_H_) and expressed as a percentage of R_S_ (i.e. %R_A_ =  (R_S_–R_H_)/R_H_*100). To estimate the contribution of R_A_ across the entire sampling period, the equation was applied to the CO_2_ flux data, together with the sediment surface temperature for each control chamber at each sampling time. The mean R_A_ contribution across the sampling period was then compared with that obtained from the final sampling time.

The total additional C emissions due to treatment (treatment-induced C emissions) were estimated as the area under the response curve based on the trapezoidal rule [50]:
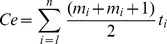
where C_e_ =  treatment induced C emissions, n =  number of measurements, m =  individual measurements and t =  time difference between any two consecutive measurements.

The difference in the rates of root decomposition in the control and treated plots was tested using a two-sample t-test. Sediment carbon concentration (g C cm^−3^) was calculated as the product of bulk density (BD) and % organic C of the sediment. Thereafter, the sediment carbon stocks down to a depth of 100 cm for each treatment were calculated as the product of carbon concentration and the depth and expressed as t C ha^−1^: C_S_ = C_C_ * (100+E_c_), where, C_S_ =  sediment C stocks, C_C_ =  C concentration and E_c_ =  elevation change. Since the control plots gained 1.1 cm and the treated plots lost 5.1 cm in surface elevation (*see* Figure 2), the depth for each treatment was adjusted to reflect these changes, i.e. 100+E_c_ = 101.1 and 94.9 cm for control and treated plots, respectively.
