# Correction: Rapid Losses of Surface Elevation following Tree Girdling and Cutting in Tropical Mangroves

**DOI:** 10.1371/journal.pone.0118334

**Published:** 2015-03-18

**Authors:** The PLOS ONE Staff

There is an error in affiliation 7 for author Maurizio Mencuccini. Affiliation 7 should be: ICERA at Centre for Ecological Research and Forestry Applications, Universitat Autònoma de Barcelona, Barcelona Spain.
